# Evaluation of PCR-RFLP in the Pre-S Region as Molecular Method for Hepatitis B Virus Genotyping

**DOI:** 10.5812/hepatmon.11781

**Published:** 2013-10-15

**Authors:** Rim Ouneissa, Olfa Bahri, Ahlem Ben Yahia, Henda Touzi, Mohamed Msaddak Azouz, Nabyl Ben Mami, Henda Triki

**Affiliations:** 1 Laboratory of Clinical Virology, Institute Pasteur de Tunis, Tunis, Tunisia; 2 Department of Gastroenterology, Hospital of Tahar Maamouri, Nabeul, Tunisia; 3 Department of Gastroenterology, Hospital La Rabta, Tunis, Tunisia

**Keywords:** Hepatitis B Virus, Genotype, Restriction Fragment Length Polymorphism, Direct Sequencing

## Abstract

**Background:**

Hepatitis B virus (HBV) infection is a public health problem in developing countries. HBV genotypes play major role in the evolution of infection since they were involved in different clinical presentations and response to treatment.

**Objectives:**

This study was conducted to evaluate the efficiency of restriction fragment length polymorphism (RFLP) analysis for HBV genotyping.

**Patients and Methods:**

We investigated 98 samples collected from patients chronically infected with HBV. HBV genotypes were determined by analysis of patterns obtained after amplification in Pre-S region and digestion of the amplicon by two endonucleases AvaII and DpnII. Obtained results were confirmed by partial sequencing in the same region.

**Results:**

Two different HBV genotypes were detected in this study, Genotype D (in 95. 9%) and Genotype A (in 4.1%). Seventy-four samples (75.5%) were successfully genotyped with RFLP analysis and all classified as genotype D. The remaining 24 samples (24.5%) which were un-genotyped by RFLP analysis, were classified by partial sequencing of the pre-S region as HBV genotype D (20 samples, 20.4%) and genotype A (4 samples, 4.1%). Atypical profiles were significantly associated with advanced liver disease (P = 0.001) as well as older age (P < 0.05).

**Conclusions:**

Several previous studies used PCR-RFLP to genotype HBV; however, we showed the high risk to obtain atypical profiles, especially in advanced stages of chronic infection, with as results difficulties to genotype the virus. These profiles resulted from the accumulation of mutations during natural course of infection resulting in a modification in restriction sites for enzymes. So, we recommended completing the investigation by partial sequencing to confirm obtained results.

## 1. Background

Hepatitis B virus (HBV) infection is the most common cause of chronic hepatitis disease with high risk of developing cirrhosis and hepatocellular carcinoma (HCC) (1). Compared to other conventional DNA viruses, HBV is characterized by complexity of its replication and high degree of genetic variability caused by an intermediate reverse-transcription step and a high level of viral releasing (1011 virions/day). Because of the lack of a 3'-5' exonuclease activity, HBV DNA polymerase generates multiple and uncorrected errors with as results multiple mutations in the entire genome and particularly in S gene. This genetic variability promotes identification of eight genotypes (A to H) based on a sequence divergence more than 8% in the entire genome, or than 4% when only the S region is considered (2-4). In addition to their different geographical distribution, HBV genotypes are also associated with different clinical outcomes and responses to antiviral treatments (5, 6). In fact, compared to genotype A, chronic infections by genotype D and C were more severe with increased risk of HCC (7-9), high risk for HBV reactivation, and high mortality rate after liver transplantation (10). In addition, low response rate to treatment with interferon-α was observed in genotype D compared to genotype A or B (8). Therefore, HBV genotyping becomes an important marker to better understanding of infection pathogenesis and prognosis (11, 12). Advances in molecular biology lead to development of several molecular methods for HBV genotyping associated with advantages and disadvantages. Sequencing of the whole genome is considered as gold standard because of its high reliability and precision (3); however, its high cost and time-consuming status limit its routine usage. The type-specific primers amplification and the line probe assay (INNO-LiPA) take less time but they are not suitable for large-scale surveys nor accurate to identify mixed infection (13, 14). To solve these problems, genotyping with restriction fragment length polymorphism was developed to distinct between HBV genotypes by profiles analysis obtained after digestion by restriction enzymes (15-17). Nowadays, this method is widely used for epidemiological studies especially in developing countries (18). Nevertheless, limited data were reported about its efficiency.

## 2. Objectives

The main purpose of this study was to assess the performance of Polymerase chain reaction-restriction fragment length polymorphism (PCR-RFLP) for HBV genotyping in comparison with partial sequencing. The correlation between unexpected profiles by RFLP and clinical status or viral load was also studied.

## 3. Patients and Methods

### 3.1. Patients

Sera were collected from 98 patients chronically infected by HBV who attended two departments of gastroenterology, in La Rabta Hospital at Tunis and Tahar Maamouri Hospital at Nabeul (a coastal region in Tunisia). All sera were tested in advance by a commercial real time PCR (COBAS TaqManTM 48 Analyzer, Roche Diagnostics, Mannheim, Germany) to evaluate HBV DNA levels; detection limit for this method was 6IU/mL and quantitation range was 6 to 1.1-108 IU/mL. Studied patients were 65 males and 33 females aged from 16 to 71 years with a mean age of 40.12 years. Informed consent was obtained for each patient enrolled in the study. This work was approved by Ethics Committee of Tunisian Ministry of Health.

### 3.2. HBV DNA Extraction and Amplification in the Pre-S Gene

HBV DNA was extracted from 200 µL serum using a QIAamp DNA Blood Mini Kit (Qiagen Inc., Hilden, Germany) as recommended by the manufacturer. Pre-S region spanning from nt 2823 to 80 was amplified, as described previously by Lindh et al. 1998, using primers P1 (5'- TCACCATATTCTTGGGAACAAGA-3', nt 2823-2845) and P2 (5'- TTCCTGAACTGGAGCCACCA -3', nt 80-61). Amplified products were used for genotyping through two different approaches; restriction fragment length polymorphism (RFLP), and partial sequencing of the surface S gene (Pre-S1 and Pre-S2 regions).

### 3.3. HBV Genotyping by RFLP Analysis of Pre-S Gene

Amplified product was digested by two restriction enzymes (AvaII and DpnII) (New England, Biolabs, Sigma-Aldrich), according to the protocol proposed by Lindh et al. 1998 (16). This method was known to detect genotypes A to F of HBV. Its sensitivity was previously estimated to be less than 103 copies/mL. Digested products were revealed on 3% agarose gel stained with ethidium bromide. HBV genotype identification was made by comparing the obtained profiles with those proposed by the same author (16).

### 3.4. HBV Genotyping by Partial Sequencing in the PreS-gene

Partial sequencing was performed for all samples to verify obtained results by PCR-RFLP. Sequencing of PCR products was done by ABI Prism 3130 Genetic Analyzer and a BigDye Terminator V.3.1 Ready Reaction Cycle Sequencing Kit. It was performed bi-directionally using the same primers than amplification. Obtained sequences were submitted to GenBank, and can be retrieved under accession numbers (KF414979- KF415076). Clustal X and BioEdit software were used for multiple alignment and comparison of obtained sequences with eight sequences representative of the major HBV genotypes retrieved from GenBank database under the following accession numbers: genotype A (M57663), B (D00330), C (X04615), D (X02496), E (X75657), F (X75658), G (AF160501), and H (AY090457). Phylogenetic tree was carried out using Molecular Evolutionary Genetics Analysis (MEGA4.1) software, and established using neighbor-joining (N-J) method and 1000 bootstrap replicates to confirm the reliability of the tree.

### 3.5. Statistical Analysis

SPSS Version 13.0 was used for all statistical analysis. χ^2^, Fisher’s exact; Chi square tests were used to assess the statistical significance of differences between studied groups. P values below 0.05 were considered statistically significant. The HBV DNA levels were analyzed by descriptive statistics such as mean range, and comparison was performed using Mann-Whitney U test.

## 4. Results

### 4.1. Patients

The 98 patients, included in this study, belonged to three different clinical groups: inactive carriers (IC, n = 14), patients with chronic active hepatitis (CAH, n = 52), or patients with a progressive liver disease (PLD, n = 32); among the latter group, 31 cases had a liver cirrhosis and one an HCC. HBV DNA levels were previously measured for all patients using real-time PCR (HBV TaqMan, Roche), ranged from 1, 4.102 to 5, 7.1010 copies/mL; the mean range was 1, 4.109 copies/mL. IC of HBsAg carriers were characterized by persistently normal ALT levels, absence of HBeAg marker, and serum HBV DNA levels below 104 copies/mL, whereas CAH was defined by persistent ALT elevation and detectable serum HBV DNA. PLD was characterized by the presence of cirrhosis (diagnosis by clinical and/or ultrasonographic signs of portal hypertension) and/or hepatocellular carcinoma (diagnosed by imaging showing the characteristic features of HCC and/or, when possible, histological assessment of tissues samples, and serum alpha-fetoprotein levels). The demographic, clinical, and virological characteristics of the patients are shown in Table 1. 

**Table 1.** Demographic, Biochemical and Virological Characteristics of Studied Population

	IC ^a^ (n = 14)	CAH ^a^ (n = 52)	PLD ^a^ (n = 32)
**Age, y** ^a^	35.1 ± 14.3	37.8 ± 12	45.8 ± 12.2
**Sex (M/F)**	8/6	26/26	31/1
**ALT ** ^**a**^ ** (IU/L)**	24.5 ± 6.49	102.7 ± 123.9	90 ± 171.5
**AST ** ^**a**^ ** (IU/L)**	24.8 ± 9.28	72.7 ± 69.2	63.3 ± 31
**Alkaline phosphatase (IU/L) ** ^**b**^	152.8 ± 49.3	193.6 ± 80.3	236.5 ± 125.2
**Total Bilirubin (µmol/L) ** ^**b**^	17 ± 8.76	18.4 ± 27.8	31 ± 34.5
**GGT (IU/L) ** ^**b**^	20.5 ± 13	65 ± 96.4	371.2 ± 1042.8
**HBV DNA levels (copies/mL) ** ^**b**^	7.9 10^7^ ± 2.1 108	2.2 10^9^ ± 8.3 10^9^	6.1 10^8^ ± 1.8 10^9^
**Fibrosis score**	F0-F1	F2-F3	F4
**HBsAg (+/-)**	14/0	52/0	32/0
**HBeAg (+/-)**	0/14	8/44	8/24
**Anti-HBe (+/-)**	13/1	44/8	22/10
**Anti-HBc (+/-)**	14/0	52/0	32/0

^a^ Abbreviations: IC, Inactive carriers; CAH, active chronic hepatitis; PLD, Progressive liver disease; ALT, alanine transaminase (international unit per liter); AST, aspartate transaminase (international unit per liter)

^b^ Mean ± SD

### 4.2. Determination of HBV Genotypes with RFLP

HBV genotype was successfully identified by RFLP analysis for 74 patients (75.5%): Genotype D was identified in all samples on the basis of criteria used by Lindh et al (16). Three typical restriction profiles were observed: D2 pattern (uncut with AvaII and three bands 306, 88b, and 52bp with DpnII) was found in 71 cases (96.0%). For two patients (2.7%), a profile, known as D1 pattern and characterized by one band of 446 bp with AvaII and four bands 306, 67, 52, and 21 bp with DpnII, was detected. Only one patient (1.3%) showed a D-del pattern corresponding to one band at 263bp with AvaII and three bands at 123, 88, and 52 bp with DpnII. For the remaining 24 samples (24.5%), atypical patterns were observed with a failure to identify genotype (Table 2). 

**Table 2.** Obtained Profiles and Accordance between Expected and Noticed Restriction Profiles for 24 Samples which were un-genotyped by RFLP

Genotype	Expected restrictions profiles resulting from digestion with		Noticed restriction profiles ^a^ resulting from digestion with		No. of each noticed profile
	DpnII	AvaII	DpnII	AvaII	
**Genotype D**	(306-88-52)	446	(306-88-52)	(145-301)	7
	(306-88-52)	446	(306-88-37-15)	(145-301)	1
	(306-52-88)	446	(358-88)	446	3
	(123-52-88)	263	(175-88)	263	1
	(306-52-88)	446	(358-88)	(145-301)	1
**Genotype A**			(306-88-40)	434	2
			(306-88-31)	425	2
	(306-88-52)	446	(213-88-52)	353	1
			(278-88-26)	392	1
			(303-88-43)	434	1
	(318-109-52)	(301-121-57)	(318-88-21-52)	(301-121-57)	3
	(318-109-52)	(358-121)	(318-88-21-52)	(358-121)	1

^a^ Noticed restriction profiles: atypical restriction profiles

### 4.3. Confirmation of Results of RFLP Analysis by Partial Sequencing

**Figure 1.**
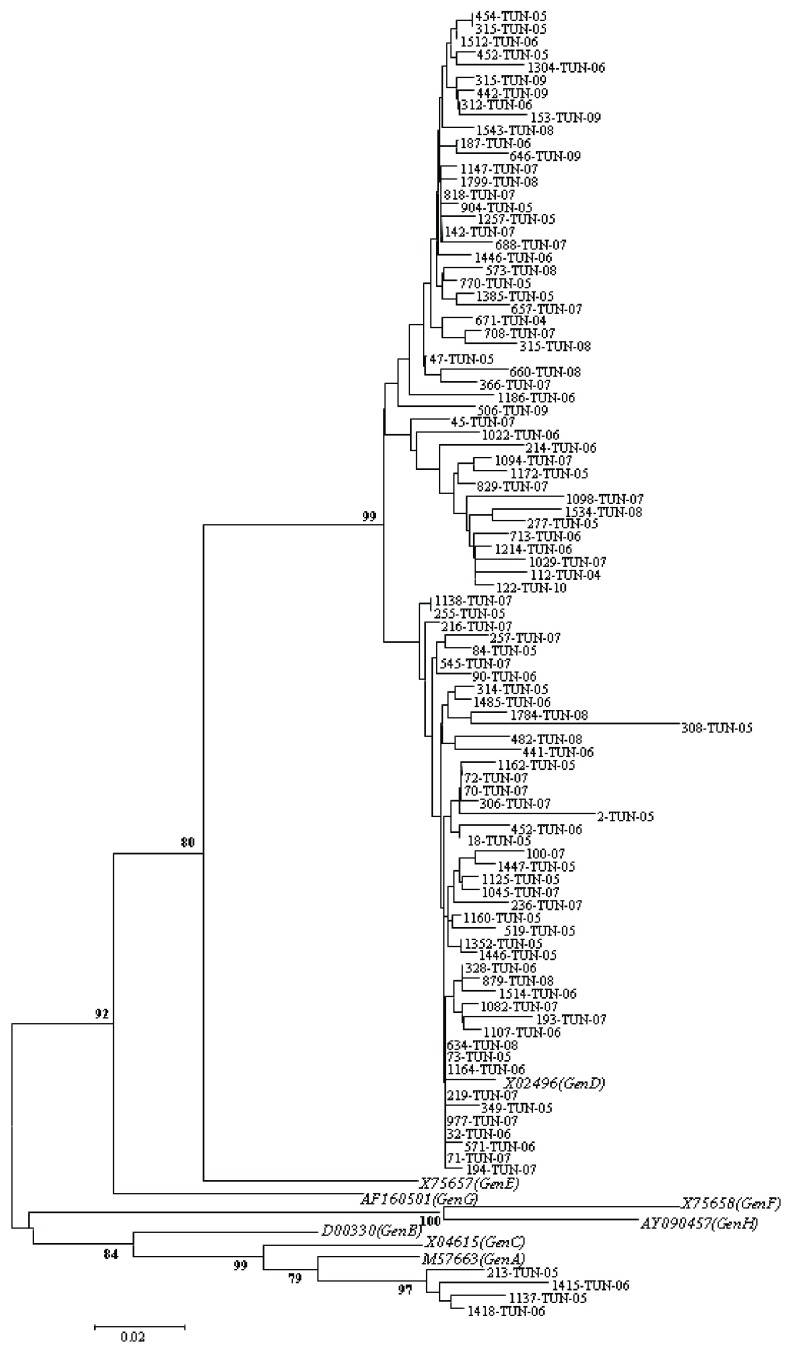
Phylogenetic Tree Based on the Analysis of a 328-bp Fragment in pre-S Gene The tree includes eight reference sequences representative of genotype (A-H) and 95 Tunisian sequences from those studied in this work. Sequences of this work were indicated by the laboratory code followed by the country code (TUN) and the year of isolation. The reference sequences were indicated by their GenBank Accession number followed by the genotype designation.

Partial sequencing was performed for all samples to verify obtained results by PCR-RFLP; it confirmed infection by genotype D for 74 samples having typical profiles by RFLP (samples characterized by D1, D2, and D-del pattern). For 24 remaining isolates which were un-typeable isolates, genotype D and A were observed in 20 and 4 cases, respectively. Overall, prevalence of two detected genotypes was 96% (Genotype D) and 4% (Genotype A). Figure 1 shows a phylogenetic tree obtained from GenBank after comparison with selected sequences. 

In un-typed samples by RFLP analysis, nucleotide sequence was studied to search for an eventual modification in restriction sites by digesting enzymes used to explain atypical profiles. An addition of one and/or two restriction sites was observed in 50% of cases (n = 12/24) and a punctual disappearance of the site in 16.7% of cases (n = 4/24). In seven cases (29.2%), a deletion of more than three nucleotides was observed. In one case (4.2%) an addition and a disappearance of at least one restriction site was detected, simultaneously. The agreement between modifications in sites of restriction and identified genotype by partial sequencing was reported in Table 2. 

### 4.4. Association of Atypical Profiles With Clinical Status, Age, and Viral Load

**Figure 2.**
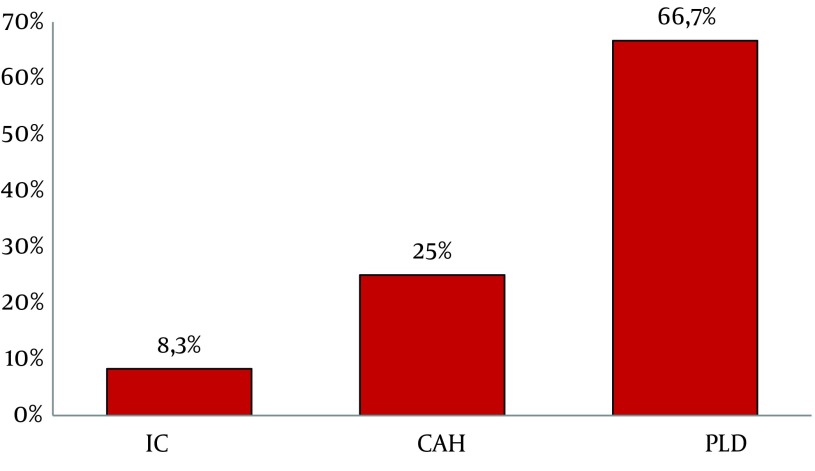
Frequency of Atypical Profiles in Each Clinical Status Ic, inactive carriers; CAH, chronic active hepatitis; PLD, progressive liver disease

Atypical patterns were highly detected in PLD group (66.7%, 16/24) compared to CAH (25%, 6/24) and IC (8.3%, 2/24) (Figure 2). These atypical profiles were also observed more frequently in aged patients ranging from 24 to 71 years old (Mean age = 47.3, P = 0.01); however, no significant association was observed with viral load. 

## 5. Discussion

In the current study, the most common genotype was genotype D which was detected in 96% of cases. These results are in agreement with previous data derived from HBV infected patients originating from Tunisia, confirming a predominance of genotype D in the country (19, 20). Genotype D prevails in all Mediterranean regions; it was reported in more than 50% of HBV infected patients from south of Europe (21). It seems that the genotype D is predominant in some other countries from the Maghreb like Morocco and Algeria which was observed in more than 87% and 93% of studied cases, respectively (18, 22-24). In other genotypes and in accordance to what was described previously, genotype A was identified in only 4% of studied samples; so it can be considered as one of the occasional identified genotypes in the country (19, 20).

Herein, the genotyping method used was a PCR-RFLP; compared to sequencing, this method is known to be relatively simple, fast, and not too expensive (25, 26). When performed in the Pre-S region, a PCR-RFLP allowed a detection for some genotypes easier than other techniques, in particular for genotype D (16). This genotype is known to have a specific 33-nucleotide deletion in the Pre-S1 region allowing generation of a specific amplicon of 446pb instead of 479pb expected and observed for all other genotypes (27, 28). Furthermore, strains from genotype D lacked AvaII restriction site, giving also a characteristic uncut band of 446 bp after AvaII digestion. All these factors make a PCR-RFLP in the Pre-S region so useful for genotype classification. So, this method was used largely throughout the world especially in regions where genotype D was predominant (18, 29, 30). In fact, the most of Tunisian and Moroccan studies performed this method successfully to identify circulating genotypes (20). Moreover, to distinguish between genotypes, when PCR-RFLP is performed in Pre-S region which is small in size, , less restriction enzyme is required. .

In the current study, PCR-RFLP allowed easily the distinction between genotypes D and A in studied population according to published determinative patterns (16). In fact, in this study, three different patterns (D1, D2, and D-del) were obtained for genotype D with predominance of D2. These results are in agreement with previous Tunisian studies where the profile D2 was the most prevalent (20). This was also the most common pattern in Morocco and Turkey which was detected in 100% and 85.9% of studied population, respectively (18, 30). It seems that RFLP patterns correlated directly with the prevalence of HBV circulating sub-genotypes, since D2 pattern prevails in countries of Maghreb where D1 and D7 were the most frequent sub-genotypes (18, 23, 31). However, this profile seems to be less prevalent in South Africa and Somalia where D3 and D4 sub-genotypes are predominant, respectively (32). In other countries, a co-circulation of strains with at least two different sub-genotypes, at comparable proportion, was shown. For example, in India four D sub-genotypes were described; D1 was found in 17%, D2 in 29%, D3 in 34%, and D5 in 20% of studied population (33). The multiplicity of patterns for the same genotype with variability in their geographical distribution should be taken into consideration especially in countries where only one genotype predominantly circulates; it plays, probably, an important role in disease progression and response to antiviral therapy. Accordingly, ongoing studies are primarily interested in molecular aspects of genotype D sub-genotypes and mutations in different regions of the genome rather than comparison between different genotypes to understand the evolution of chronic hepatitis B (34).

In this study, a correct genotyping rate for PCR-RFLP was important, since partial sequencing confirmed the results obtained from all samples with typical profiles. For that, we can consider PCR-RFLP a suitable and appropriate applicable screening method for HBV genotyping. However, this technique failed to identify the genotype in 24.5% of cases making investigation compulsorily to be completed by another method, such as partial sequencing, if applicable. Atypical patterns were also reported by previous studies using the same method with rates varying from 2.5 to 22.2% (29, 30, 35-38). All these studies classified these patterns as genotype D when investigation was completed by direct sequencing. This disadvantage was also reported for other methods such as PCR-hybridization or type-specific primers amplification, and also for other HBV genotypes (29, 30, 35-40). The limit to genotyping in all these cases was probably due to high variability of HBV with modification in expected restriction profiles for used enzymes (41). Current results confirmed that hypothesis since 24 atypical profiles were detected; they were explained by either adding one or more restriction sites or disappearing at least one restriction site (41). These atypical profiles were observed especially after a long evolution of the disease, since they were significantly associated with advanced age and PLD rather than groups of CAH and IC. These findings support previous reports about chronic HBV infection, which was characterized, in its ultimate stages, by an accumulation of mutations under host immune pressure (34, 42-46). Finally, atypical profiles could be explained by inability of the method used in this study to distinguish between eight sub-genotypes described for Genotype D (31) or to detect genotypes G and H (16). However, this hypothesis is very unlikely because, up to now, there was no report of circulation for these two genotypes, neither in the country nor in most of Mediterranean countries. In fact, these two genotypes were known to have a limited geographical circulation. Genotype G was known to be circulating in some European countries (4) and in North America (11, 47), and genotype H in Central America (11, 48). In conclusion, the results from this studied population suggest that RFLP, which was frequently used for HBV genotyping in a routine clinical virology laboratory setting, has some additional limitations. They are raised from atypical profiles observed especially in advanced stages of chronic infection. Heterogeneous sequences of sample populations may cause problems in the genotyping using only one method. In these cases both methods and supplemental tests might be necessary.
